# The impact of *Elaeagnus angustifolia* root exudates on *Parafrankia soli* NRRL B-16219 exoproteome

**DOI:** 10.7150/jgen.93243

**Published:** 2024-05-11

**Authors:** Ikram kammoun, Guylaine Miotello, Karim Ben Slama, Jean Armengaud, Faten Ghodhbane-Gtari, Maher Gtari

**Affiliations:** 1 Department of Biological and Chemical Engineering USCR Molecular Bacteriology and & Genomics, National Institute of Applied Sciences and Technology, University of Carthage, Tunis, Tunisia.; 2 Département Médicaments et Technologies pour la Santé (DMTS), CEA, INRAE, Université Paris-Saclay, SPI, 30200 Bagnols sur Cèze, France.; 3 Higher Institute of Applied Biological Sciences, Laboratory of Bioresources, Environment, and Biotechnology, University of Tunis El Manar, Tunis, Tunisia.; 4 Higher Institute of Biotechnology of Sidi Thabet, University of La Manouba, Sidi Thabet, Tunisia.

## Abstract

Root exudates from host plant species are known to play a critical role in the establishment and maintenance of symbiotic relationships with soil bacteria. In this study, we investigated the impact of root exudates from compatible host plant species; *Elaeagnus angustifolia* on the exoproteome of *Parafrankia soli* strain NRRL B-16219. A total of 565 proteins were evidenced as differentially abundant, with 32 upregulated and 533 downregulated in presence of the plant exudates. Analysis of the function of these proteins suggests that the bacterial strain is undergoing a complex metabolic reprogramming towards a new developmental phase elicited in presence of host plant root exudates. The upregulation of Type II/IV secretion system proteins among the differentially expressed proteins indicates their possible role in infecting the host plant, as shown for some rhizobia. Additionally, EF-Tu, proteins upregulated in this study, may function as an effector for the T4SSs and trigger plant defense responses. These findings suggest that *Parafrankia soli* may use EF-Tu to infect the actinorhizal host plant and pave the way for further investigations of the molecular mechanisms underlying the establishment of symbiotic relationships.

## Introduction

*Parafrankia* is a bacterial genus classified within the* Frankiaceae* family, alongside the genera* Frankia, Protofrankia,* and* Pseudofrankia* 1, 2*. Parafrankia* strains are known for their ability to form symbiotic relationships with actinorhizal plants of the *Elaeagnaceae*, *Colletieae* (*Rhamnaceae*), *Morella* (*Myricaceae*), and *Gynmnostoma* (*Casuarinaceae*) species. Within the formed root nodules, the bacteria are able to fix atmospheric nitrogen, which can then be utilized by the plant as a nutrient source. The rhizosphere serves as the site for the symbiotic signalling cascade, which coordinates the regulation of genes and exchange of symbiotic signals 3. This intricate process leads to mutual recognition and, ultimately, the formation of functional root nodules. Although actinorhizal and legume root nodules share many developmental characteristics 4, there are notable differences in certain molecular signals.

In legume symbiosis, flavonoids have been identified as crucial signalling molecules during the early stages 5. These compounds act as chemotactic signals for rhizobia and specifically bind to the rhizobial NodD protein. As a result, this protein activates the transcription of nodulation genes essential for the synthesis of lipochito-oligosaccharide (LCO) Nod factors 6, 7. Subsequently, these Nod factors transmit signals back to the host plant by binding to LysM receptor kinases, initiating the activation of the common symbiotic signalling pathway (CSSP). The CSSP is a shared signalling pathway found in both legume symbiosis and mycorrhizal symbiosis 8. While there is a belief that actinorhizal plants also employ flavonoids as signalling molecules, there is currently a lack of direct evidence to support their role in the process 9-14. In almost all frankia genomes, canonical nod genes are generally absent 15, 16. Furthermore, there is no indication that early frankia signalling relies on canonical nodABC genes or molecules associated with rhizobial Nod factors, even when these genes are present in their genomes 17, 18. The conserved symbiotic signalling pathway (CSSP) is also involved in the communication actinorhizal plant and their frankia microsymbionts 19, 20. Typically, receptor complexes with LysM motifs are responsible for binding GlcNAc-based elicitors such as chitin, chitin oligosaccharides with lipid modifications; Myc factors 21 and Nod factors 22, and peptidoglycan 23-25 which consist of alternating GlcNAc and N-acetylmuramic acid residues linked by peptides. LysM-receptor-like kinases can also detect proteinaceous elicitors like flg22 from flagellin and nlp20 26-28, a conserved epitope found in bacteria, fungi, and oomycetes 29, 30. There are other receptor classes involved in recognizing lipopolysaccharides (lectin-like) 30. Secretion systems have been demonstrated to play crucial metabolic roles in exporting various molecules including effectors which are instrumental in manipulating host cellular processes and also in sensing and responding to changes in the environment, particularly within the context of plant symbiotic bacteria 31, 32.

Studies have explored the cellular proteomes of *Frankia alni*, *Protofrankia coriariae*, and *Parafrankia soli* species following treatments with host root exudates in order to describe the induced molecular dynamics 18, 33, 34. The results of these studies indicate that the symbiotic signalling systems in actinorhizal symbiosis are highly intricate and tightly regulated. According to a study conducted by Gueddou et al. 18, proteins involved in various biological processes showed increased expression when exposed to root exudates from *Elaeagnus angustifolia*. These proteins are associated with nitrogen fixation and assimilation, respiration, oxidative stress, proteolysis, and plant cell wall degradation. Thus far, there have been no significant findings of any candidate proteins linked to nodulation factors that can be sensed by LysM-receptor-like kinases and leading ultimately to a signal transduction cascade in actinorhizal plants.

The bacterial exoproteome is the entirety of proteins that a bacterial cell releases into the environment through secretion systems, outer membrane vesicles, or lysis 35; 36. The proteins within the bacterial exoproteome have diverse functions in bacterial physiology 36-39. This can include proteins that aid in nutrient acquisition, such as transporters and enzymes that break down complex molecules 40. Additionally, the exoproteome can contain proteins that bind to host or microbial receptors, which allows for the mediation of signalling 41; 42. Quorum sensing molecules are also present in the exoproteome and allow bacteria to coordinate their behaviour based on population density 39; 43; 44. Some proteins within the exoproteome can also modulate the host immune system, suppressing host defences 39; 45 or promoting the growth of host tissues 46-48.

In the early stages following plant stimuli, it has been shown that rhizobial exoproteome comprises adhesins that assist in bacterial attachment to roots, enzymes necessary for the modification of surface polysaccharides, and effectors that can either suppress plant defense responses or activate specific signalling pathways 7.

In this study, we present the extracellular proteome analysis of *Parafrankia soli* strain NRRL B-16219, which was treated with root exudates in a minimal medium. The goal was to identify whether the secreted proteins during the early response phase of *Parafrankia soli* strain NRRL B-16219 to plant stimuli contained a significant amount of symbiotically relevant proteins and could provide insights into symbiotic signalling.

## Materials and Methods

### Production of root exudates

To obtain root exudates,* Elaeagnus angustifolia* seedlings were grown axenically in Broughton and Dilworth 49 nutrient solution supplemented with 5 mM KNO3 as the nitrogen source (BD+N). Two weeks later, the BD+N medium was replaced with nitrogen-free BD medium (BD-N and root exudates were collected after two additional weeks seedling growth. The collected exudates underwent filter sterilization using a 0.22 µm polycarbonate membrane.

### Bacterial growth conditions and protein extraction

*Parafrankia soli* strain NRRL B-16219 50 was cultivated in 125 ml bottles containing 40 ml of Broughton and Dilworth solution without nitrogen (BD-N), supplemented with 5 mM pyruvate as the carbon source, at a temperature of 28°C without shaking. After five days of exponential growth, one volume (v/v) of freshly collected root exudate was introduced to the culture. In control experiments, one volume of BD-N was added to NRRL B-16219 cultures grown under identical conditions. Following three days of exposure to the root exudates, the exoproteomes of NRRL B-16219 were analysed using the methods described previously 34. Each experiment consisted of four independent biological replicates.

### Nano-liquid chromatography and tandem mass spectrometry analysis

To analyse the peptide digests, we employed an Ultimate 3000 LC system (Thermo-Scientific, Villebon-sur-Yvette, France), following the detailed protocol outlined in Ktari et al. 34 and subsequently in Gueddou et al. 18. The MS/MS spectra were examined using the MASCOT 2.3.02 search engine (Matrix Science, London, UK) with standard parameters, as described by Hartmann and Armengaud (2014). The search was conducted against the complete list of annotated CDS from the draft genome of *Parafrankia soli* strain NRRL B-16219 (GenBank/EMBL/DDBJ accession number MAXA00000000.1), which comprises 6,679 protein sequences 17. Peptide matches exceeding the peptidic identity threshold were filtered based on a significance level of P < 0.05. Validated proteins were those that had at least two peptide sequences assigned to them, following the principle of parsimony. For protein abundance evaluation, we employed a previously described approach 51; 52 involving shotgun analysis with MS/MS spectral counts. The calculation of normalized spectral count abundance factors was performed following the methodology outlined by Paoletti et al. 53. The resulting values were expressed as percentages of the total signal.

### Data analysis

Computational predictions of protein subcellular localization data were performed based on Subcellular localization of proteins was predicted with PrediSi software 54. Signal peptide sequences were further investigated at the CBS prediction server (http://www.cbs.dtu.dk./services/), using SignalP version 6.0 55, TatP version 1.0 56, and SecretomeP version 2.0 57.

Type IV secretion system proteins were identified by T4SEpre (beta) 58 which predicts Type IV secreted proteins based on amino acid composition in C-termini and using EffectiveDB 59 with the plant classification module and selective (0.5) restriction value method enabled.

Differentially detected proteins were categorized into functional classes and re-annotated using FUNAGE-Pro v1 software 60. FUNAGE-Pro also allows enrichment analysis and additionally predicts most relevant functions.

## Results

### General characteristics of *Parafrankia soli* NRRL B-16219 exoproteome

**Figure 1:**
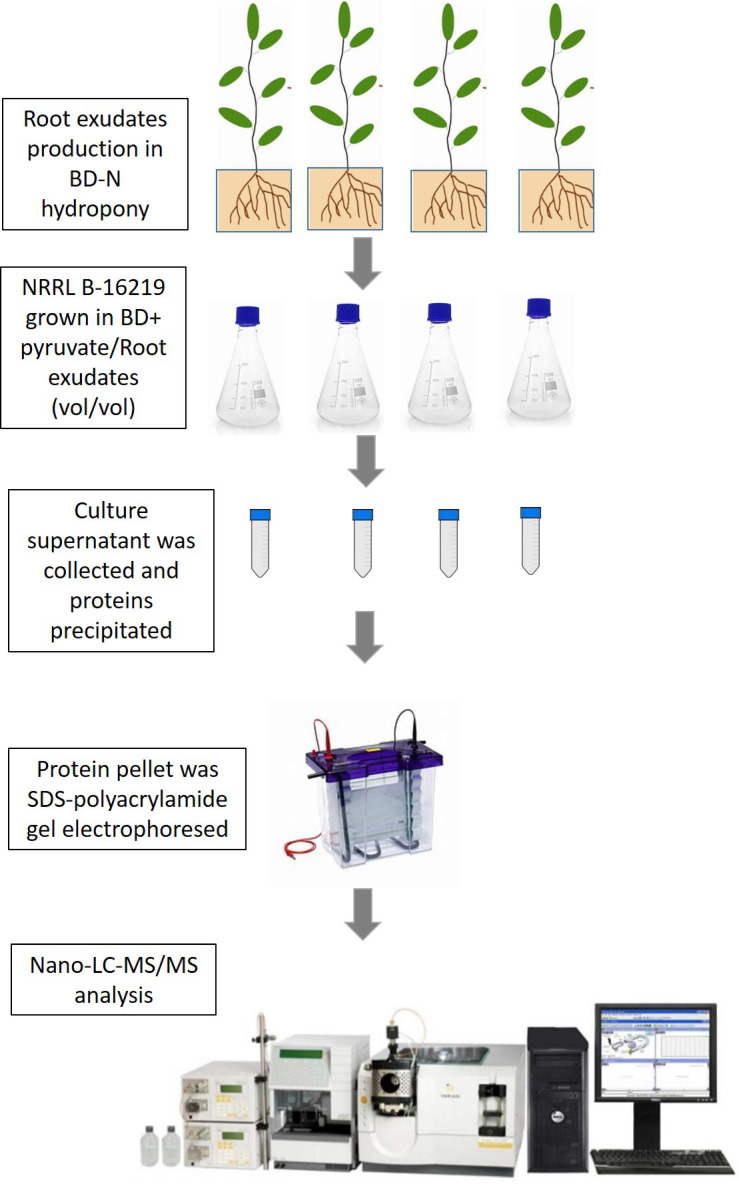
Experimental pipeline used in the present study for root exudate production, treatment of NRRL B-16219, protein precipitation and NanoLC-MS/MS analysis.

The experimental pipeline for producing root exudates, treat NRRL B-16219, and analysing the exoproteome by next-generation shotgun proteomics using high resolution nanoLC-MS/MS, was summarized in Fig. 1. As expected, the root exudate by itself was very low in terms of protein load and resulted in negligible peptide identification 18. The analysis of the exoproteome from strain NRRL B-16219 using tandem mass spectrometry generated a total of 306,055 MS/MS spectra, among which 39,216 could be confidently assigned to peptide sequences when results from all samples were combined (Supplementary Tables S1). The percentage of assignment reached 29% for the exoproteome of untreated bacteria, a ratio commonly found for other bacteria 61; 62. The experimental dataset comprised a total of 7,324 peptide sequences that were attributed to 2,011 proteins detected, with 948 of them certified by at least 2 peptides, representing the exoproteome from strain NRRL B-16219 grown in the presence or absence of root exudates. Additional information such as the abundance of each of these proteins per replicate and condition can be found in Supplementary Table S2-S3.

A threshold of ± fold (≥1.5) with a *p*-value ≤0.05 was employed to identify proteins that were differentially detected, either up- or down-regulated. Out of the 565 differential abundant proteins in the presence of *E. angustifolia* root exudates, there were 32 upregulated and 533 downregulated proteins (Supplementary table S4).

Most of differentially abundant proteins were predicted to be cytoplasmic (53.6%) followed by unknown localisation (24.2%) and membrane/cytoplasmic (17.7%). Fewer were predicted to occur in the extracellular (2.4%) or cell wall (2.1%) compartments (Supplementary Table S3).

Result for the detection of signal peptide sequences with potential cleavage site was found to be thin. Exception is for “MULTISPECIES_ substrate-binding domain-containing protein” (WP_083390861.1) putatively secreted through Sec machinery with signal peptide probability of 0.78 and probable cleavage site between 43-44 residues. The “Aspartate aminotransferase family protein” (WP_071066848.1) was predicted to route through twin-arginine translocation (Tat) pathway with a cleavage site most likely between position 46 and 47 residues.

### Functional analysis of differently expressed proteins

**Figure 2:**
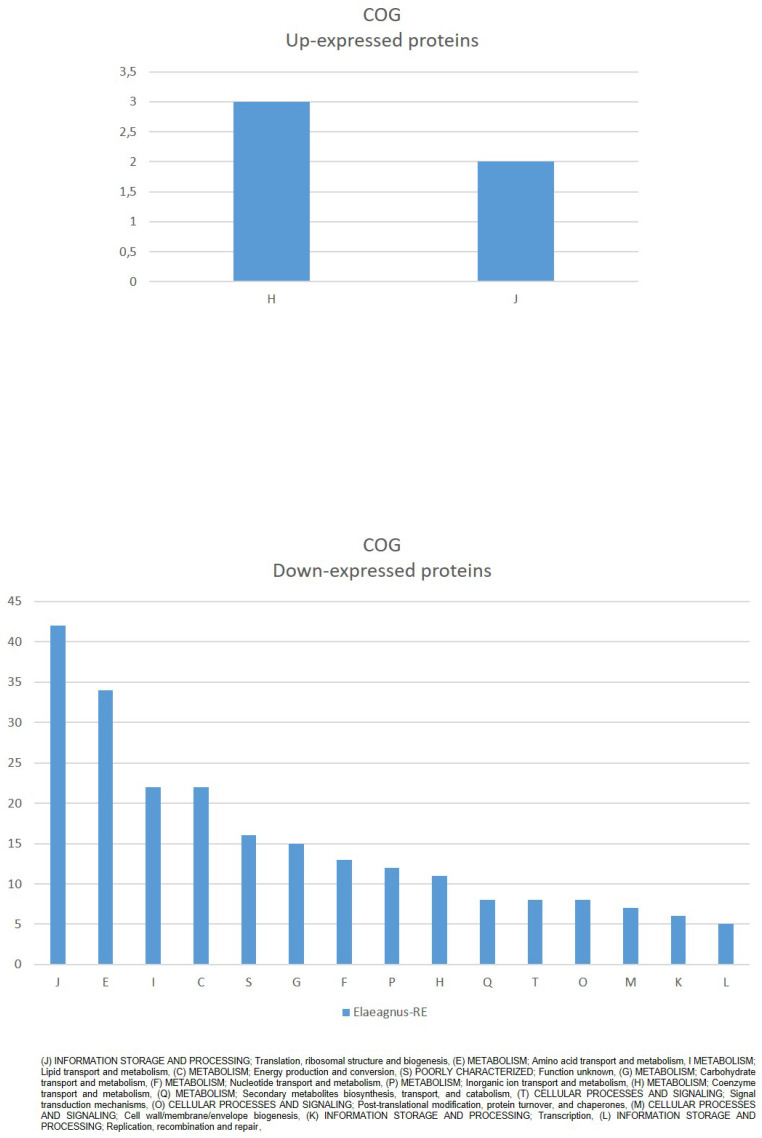

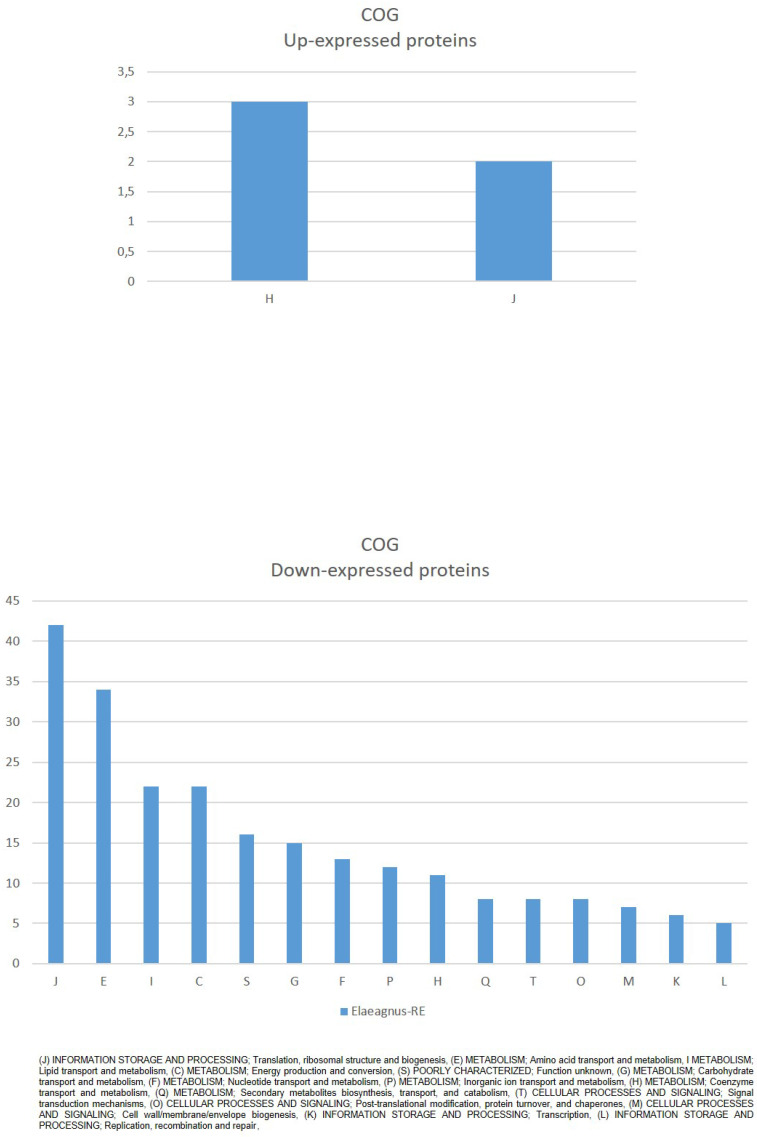
COG classification of significantly affected proteins in *Parafrankia soli* strain NRRL B-16219 exoproteome in presence of root exudates of *E. angustifolia* host species. A graphical representation of up- (a) and down-expressed proteins (*p* value ≤ 0.05, Tfold ≥ 1.5 and hits/class score = 9 (0-9, 0 being not significant, ranked based on Benjamini-Hochberg Algorithm)).

Clusters of Orthologous Groups (COG) mapping (Fig. 2) showed that down-expressed proteins were mostly (J) INFORMATION STORAGE AND PROCESSING; Translation, ribosomal structure and biogenesis, (E) METABOLISM; Amino acid transport and metabolism, I METABOLISM; Lipid transport and metabolism, (C) METABOLISM; Energy production and conversion, among others. While up-expressed proteins were assigned to (H) METABOLISM; Coenzyme transport and metabolism, and (J) INFORMATION STORAGE AND PROCESSING; Translation, ribosomal structure and biogenesis (Fig. 2).

**Figure 3:**
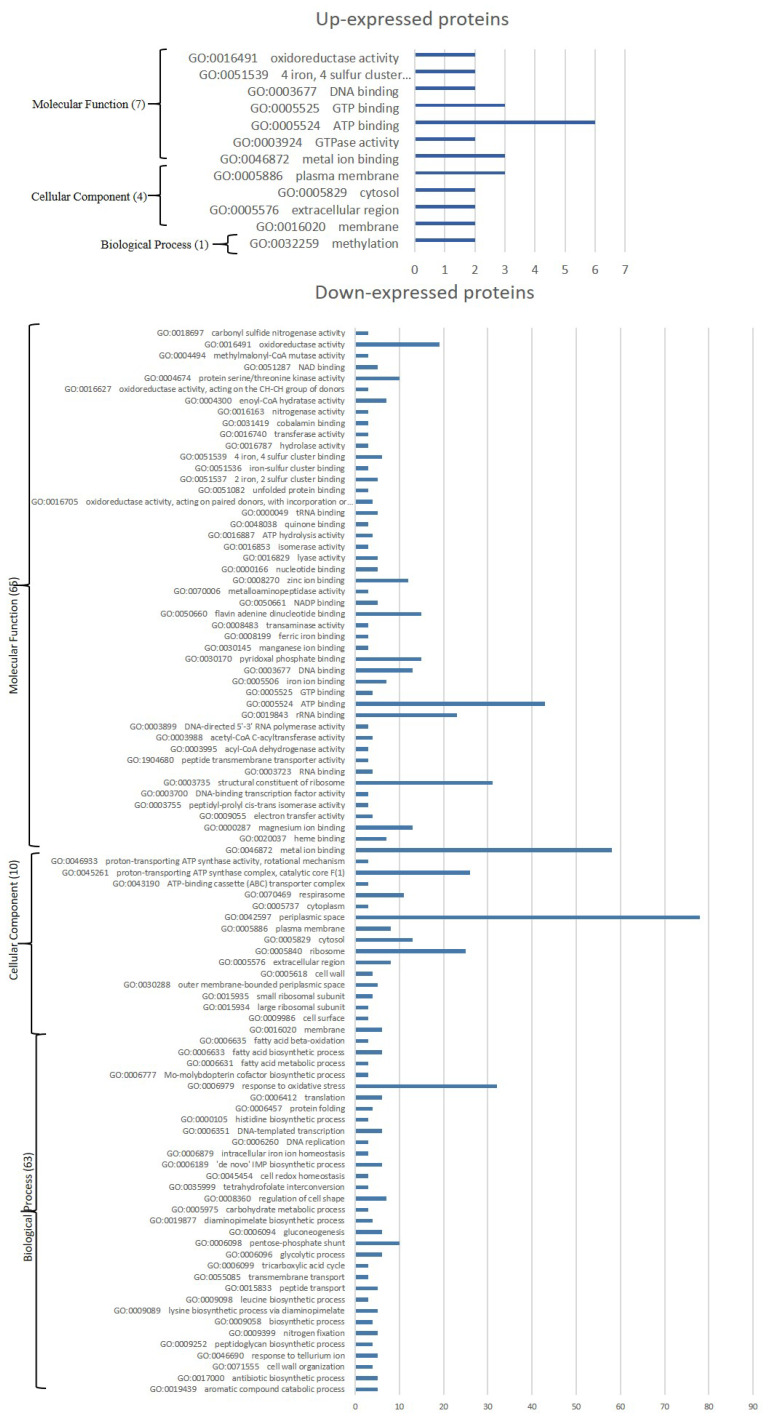
GO terms significantly affected in *Parafrankia soli* strain NRRL B-16219 exoproteome in presence of root exudates of *E. angustifolia* host species. A graphical representation of up- (a) and down-expressed proteins (*p* value ≤ 0.05, Tfold ≥ 1.5 and hits/class score = 9 (0-9, 0 being not significant, ranked based on Benjamini-Hochberg Algorithm)). Only hits/class size >2 are presented.

**Figure 4:**
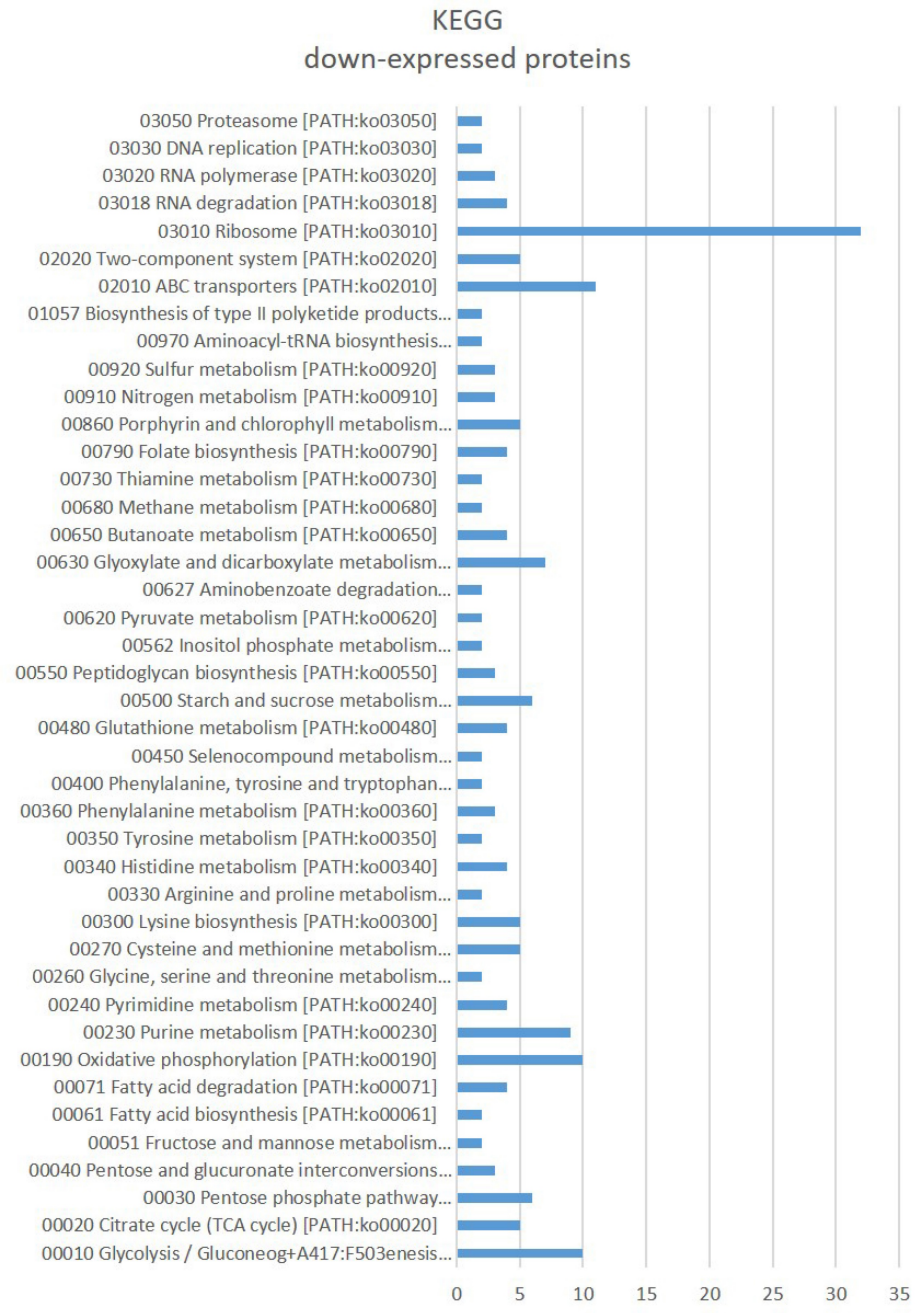
KEGG terms significantly affected in *Parafrankia soli* strain NRRL B-16219 exoproteome in presence of root exudates of *E. angustifolia* host species. A graphical representation of down-expressed proteins (*p* value ≤ 0.05, Tfold ≥ 1.5 and hits/class score = 9 (0-9, 0 being not significant, ranked based on Benjamini-Hochberg Algorithm)).

Gene Ontology (GO) enrichment (Fig. 3) assigned dawn-expressed proteins mainly to “metal ion binding”, “ATP binding”, 'structural constituent of ribosome”, “RNA binding”, 'oxidoreductase activity' among others within Molecular Function, “cytoplasm”, “plasma membrane” and 'ribosome' among others as Cellular Component, and Biological Process include “translation” and “tricarboxylic acid cycle” among others. Kyoto Encyclopedia of Genes and Genomes (KEGG) showed “Ribosome”, ABC transporters, Glycolysis / Gluconeogenesis, “Oxidative phosphorylation”, “Purine metabolism”, “Glyoxylate and dicarboxylate metabolism”, “Starch and sucrose metabolism” and 'Pentose phosphate pathway” amongst the main identified sets (Fig. 4).

GO enrichment of up-expressed proteins (Fig. 2) mainly identify “ATP binding', 'GTP binding' and “metal ion binding” among others for Molecular Function, “plasma membrane” for Cellular Component among others, and “methylation' Biological Process. Among up-expressed proteins only “Cysteine and methionine metabolism” was identified as connected to KEGG pathway.

To gain insight functional analysis up-regulated proteins were further assigned based on InterPro functional classification (Table 1). Interestingly “S-adenosyl-L-methionine-dependent methyltransferase”, “Type II/IV secretion system protein” and “Elongation factor Tu” were identified. The latter “Elongation factor Tu” was the only predicted as Type IV secretion system's effector among up-expressed proteins.

Detected enzymes are mainly Transferases (5), followed by Lyases (3), Hydrolases (2), and Oxydoreductases, Isomerases and Translocases (1 each) as indicated in Table 1.

## Discussion

The abundance exoproteins, detected in NRRL B-16219 without root exudate treatment, including a high proportion of proteins predicted to have cellular localisation, may be explained by extensive cell autolysis which is a common feature of microbial growth associated with a variety of cultural factors and stresses (Shockman et al., 1996). For frankia, studies have reported a similar trend of autolysis in both static and stirred defined medium 64-66, under nitrogen-fixing conditions 67, and even in aging nodules 68. Mastronunzio et al. 69 suggested that detected proteins in exoproteome is due to frankia cell lysis during growth rather than true secretion. There is a possibility that more secreted proteins in Frankia are present, but they might be either attached to the membrane or associated with the cell envelope, thus not being detected in the medium. In the present study the exoproteome of strain NRRL B-16219 showed a significant decrease in abundance after five days of growth when exposed to root exudates compared to the control condition. This observation suggests that the cellular autolysis may ceased indicating a potential recovery of growth. Most studies attributed carbon source depletion as a stressful condition that can trigger cellular proteolysis, which in turn can lead to autolysis 70. While in rich organic media most analysed frankia displayed long stationary phases and cells remain viable up to one year 65. Therefore, NRRL B-16219 may perceive root exudates as abundant nutrient sources, potentially postponing its cell autolysis and promoting the continuous exponential growth of the strain.

Proteins involved in autolysis consisted of proteasomes (WP_071063554.1, WP_071063556.1 and WP_071063560.1), aminopeptidase (WP_071065370.1, WP_071061455.1, WP_071059318.1, WP_071066137.1, WP_071063686.1), and peptidoglycan endopeptidase (WP_071063732.1) which may be seen as responsible for catabolism of the cell-wall under nutrient deficiency 71. Benoist et al. (1992) 70 reported that these proteinase subunits exhibited a significant increase in activity upon cessation of growth within 5 days old culture in stirred mineral medium. The addition of fresh BAP medium or carbon source (Propionate), but not nitrogen source (NH_4_Cl), at the end of the exponential growth phase extended growth for an additional day and delayed the increase in activity of the proteinase subunits for 3 days after cessation of growth. However, upon resuspending frankia cells in the late exponential phase (3 days) in a culture filtrate obtained from a 5-day-old culture and supplemented with BAP-PCM medium components, the biomass yield decreased to approximately 50%.

Another downregulated protein thought to be related to autolysis is GlcNAc-PI de-N-acetylase (WP_020458433.1), which is involved in the hydrolysis of the bacterial cell wall and signalling the need for development under nutrient-limiting conditions. According to van Bergeijk et al. 72, autolytic degradation of the cell-wall peptidoglycan releases amino sugars such as GlcNAc and N-acetylmuramic acid (MurNAc) around the colonies. GlcNAc accumulation triggers development and antibiotic production under famine conditions (signalling starvation), while it blocks both processes under feast conditions (signalling abundance) 73. Another novel concept is advanced that peptidoglycan deacylases is proposed to be seen as virulence factors 74; 75.

Among the up-expressed proteins S-adenosyl-L-methionine-dependent methyltransferase (WP_071065581.1, WP_071066904.1). Geelen et al. 76 suggested that NodS acts as a methyltransferase that depends on S-adenosyl-L-methionine and is necessary for the methylation of chitin oligosaccharides lacking acetyl groups at the non-reducing end. Because no upstream Nod proteins of NRRL B-16219 were detected in its cellular proteome 18 nor, here, in its exoproteome, this protein may act differently to what has been described for rhizobia signalling.

Bacterial type IV secretion systems (T4SSs) (WP_071064601.1, WP_071066193.1), which are up-expressed proteins, belong to the bacterial type IV secretion systems (T4SSs). This is a diverse translocation superfamily, as noted by Grohmann et al. 77, that encompasses various functions. The T4SSs are primarily composed of two major subfamilies: (i) conjugative systems that enable transfer of DNA between bacteria, and (ii) effector translocators that either inject effector macromolecules directly into prokaryotic or eukaryotic host cells or secrete them into the surrounding medium, leading to a variety of effects on host cell functions during infection 78.

It has been shown that *Mesorhizobium loti* and *Sinirhizobium meliloti* secrete via a T4SSs specific proteins that affect nodulation 79; 80. Notably, in certain rhizobia, the genes involved in nodulation factor synthesis and encoding the type IV secretion system are under the control of a common regulator that is activated by flavonoids released by root legumes 6.

Up-expressed proteins EF-Tu (WP_071059306.1, WP_071066147.1) were the only predicted in this study as effector of T4SS. EF-Tu have been detected in the exoproteomes of many microbial pathogens 81-84, where it affects calcium cycling and elicits plant defense responses such as promoting Ca2+ influx across the membrane, induction of an oxidative burst, activation of calcium-dependent protein kinases and mitogen-activated protein kinase (MAPK) cascades 81; 85. Most of these plant reactions have been observed in root epidermal cells following the infection by rhizobia 86 or frankia 87.

In conclusion, the observed alterations of the exoproteome of *Parafrankia soli* strain NRRL B-16219 in response to root exudates of *E. angustifolia* indicates that the strain is adapting to its new surroundings microniche. The differential expression of proteins indicates that the strain might be undergoing a complex metabolic reprogramming and ceasing autolysis to acquire and utilize nutrients for a new developmental phase as part of the transition on the road to symbiotic lifestyle. The identification of T4SSs among the up-expressed proteins suggests that they may play a crucial role in infecting the host plant, similar to some rhizobia which use T4SSs to positively or negatively influence nodulation. Additionally, EF-Tu, which was up-regulated in this study, could serve as an effector for the identified T4SSs. EF-Tu has been detected in numerous microbial pathogens and symbionts and has been shown to trigger plant defense responses, particularly in root epidermal cells. Hence, it is plausible that frankia employs EF-Tu effector during actinorhizal plant infection. The up-regulated S-adenosyl-L-methionine-dependent methyltransferase in NRRL B-16219, without detectable upstream Nod proteins, suggests a distinct signalling role from rhizobial nod-dependent pathways.

## Supplementary Material

Supplementary tables.

## Figures and Tables

**Table 1:** Statistically significant up-expressed proteins (*p* value ≤ 0.05 and a Tfold ≥ 1.5) categorized into functional classes and annotated by COG, GO, KEGG and InterPro analysis using FUNAGE-Pro v1 software (de Jong et al., 2022)

Class ID	Description	Score*;* Hits/Class Size*; p*-value*	
IPR000640	Elongation factor EFG, domain V-like	9; 2/2; 0.00	WP_071059306.1,WP_071066147.1
IPR029058	Alpha/Beta hydrolase fold	9; 2/2; 0.00	WP_071063096.1,WP_071066904.1
GO:0005524	ATP binding	9; 6/6; 0.00	WP_071059701.1,WP_071064601.1,WP_071064902.1,WP_071065597.1,WP_071066117.1,WP_071066193.1
IPR027417	P-loop containing nucleoside triphosphate hydrolase	9; 7/7; 0.00	WP_071059306.1,WP_071059701.1,WP_071064601.1,WP_071064902.1,WP_071065597.1,WP_071066147.1,WP_071066193.1
00270 Cysteine and methionine metabolism [PATH:ko00270]	ko00270	9; 2/2; 0.00	WP_071060170.1,WP_071064376.1
IPR031157	Tr-type G domain, conserved site	9; 2/2; 0.00	WP_071059306.1,WP_071066147.1
J	INFORMATION STORAGE AND PROCESSING; Translation, ribosomal structure and biogenesis	9; 2/2; 0.00	WP_071064582.1,WP_071066147.1
IPR009000	Translation protein, beta-barrel domain superfamily	9; 2/2; 0.00	WP_071059306.1,WP_071066147.1
IPR041095	Elongation Factor G, domain II	9; 2/2; 0.00	WP_071059306.1,WP_071066147.1
IPR000795	Transcription factor, GTP-binding domain	9; 2/2; 0.00	WP_071059306.1,WP_071066147.1
IPR036188	FAD/NAD(P)-binding domain superfamily	9; 2/2; 0.00	WP_071059950.1,WP_071066784.1
IPR022399	Helicase/secretion neighbourhood ATPase	9; 2/2; 0.00	WP_071064601.1,WP_071066193.1
IPR035647	EF-G domain III/V-like	9; 2/2; 0.00	WP_071059306.1,WP_071066147.1
GO:0005525	GTP binding	9; 3/3; 0.00	WP_071059306.1,WP_071066147.1,WP_083390659.1
GO:0032259	methylation	9; 2/2; 0.00	WP_071060170.1,WP_071066904.1
GO:0051539	4 iron, 4 sulfur cluster binding	9; 2/2; 0.00	WP_071060541.1,WP_083390659.1
GO:0003677	DNA binding	9; 2/2; 0.00	WP_071062294.1,WP_071065581.1
IPR029063	S-adenosyl-L-methionine-dependent methyltransferase	9; 2/2; 0.00	WP_071065581.1,WP_071066904.1
GO:0046872	metal ion binding	9; 3/3; 0.00	WP_071060541.1,WP_071066896.1,WP_083390659.1
IPR005225	Small GTP-binding protein domain	9; 2/2; 0.00	WP_071059306.1,WP_071066147.1
GO:0005576	extracellular region	9; 2/2; 0.00	WP_071060541.1,WP_071066896.1
GO:0005829	cytosol	9; 2/2; 0.00	WP_071060170.1,WP_071060541.1
GO:0005886	plasma membrane	9; 3/3; 0.00	WP_071060541.1,WP_071064376.1,WP_071067042.1
GO:0016491	oxidoreductase activity	9; 2/2; 0.00	WP_071059950.1,WP_071066784.1
GO:0003924	GTPase activity	9; 2/2; 0.00	WP_071059306.1,WP_071066147.1
GO:0016021	integral component of membrane	9; 2/2; 0.00	WP_071064376.1,WP_071067042.1
GO:0000746	conjugation	9; 2/2; 0.00	WP_071064601.1,WP_071066193.1
H	METABOLISM; Coenzyme transport and metabolism	9; 3/3; 0.00	WP_020463537.1,WP_071066904.1,WP_083390659.1
IPR004161	Translation elongation factor EFTu-like, domain 2	9; 2/2; 0.00	WP_071059306.1,WP_071066147.1
IPR001482	Type II/IV secretion system protein	9; 2/2; 0.00	WP_071064601.1,WP_071066193.1
Enzymes	**Oxidoreductases** ndh; NADH:quinone reductase (non-electrogenic) [EC:1.6.5.9] **Transferases** metH, MTR; 5-methyltetrahydrofolate--homocysteine methyltransferase [EC:2.1.1.13], NMT; phosphoethanolamine N-methyltransferase [EC:2.1.1.103] small RNA 2'-O-methyltransferase [EC:2.1.1.386] pks12; mycoketide-CoA synthase [EC:2.3.1.295] ubiX, bsdB, PAD1; flavin prenyltransferase [EC:2.5.1.129] mucR; diguanylate cyclase [EC:2.7.7.65] **Hydrolases** atzF; allophanate hydrolase [EC:3.5.1.54] hrpB; ATP-dependent helicase HrpB [EC:3.6.4.13] **Lyases** DDC, TDC; aromatic-L-amino-acid/L-tryptophan decarboxylase [EC:4.1.1.28 4.1.1.105] moaA, CNX2; GTP 3',8-cyclase [EC:4.1.99.22] ACO, acnA; aconitate hydratase [EC:4.2.1.3] **Isomerases** groEL, HSPD1; chaperonin GroEL [EC:5.6.1.7] **Translocases** cpaF, tadA; pilus assembly protein CpaF [EC:7.4.2.8]		WP_071066784.1 WP_071060170.1 WP_071066904.1 WP_071064742.1 WP_071063096.1 WP_020463537.1 WP_071067042.1 WP_071060056.1 WP_071064902.1 WP_071066848.1 WP_083390659.1 WP_071060541.1 WP_071066117.1 WP_071066193.1, WP_071064601.1

*The values correspond to Score (0-9, 0 being not significant, ranked based on Benjamini-Hochberg Algorithm), *p* value and the hits/class size. Not applicable for enzymes EC assignment.

## References

[1] Gtari M (2022). Taxogenomic status of phylogenetically distant Frankia clusters warrants their elevation to the rank of genus: A description of Protofrankia gen. nov, Parafrankia gen. nov, and Pseudofrankia gen. nov. as three novel genera within the family Frankiaceae. Front Microbiol.

[2] Oren A, Göker M (2023). Validation List no. 210. Valid publication of new names and new combinations effectively published outside the IJSEM. Int J Syst Evol Microbiol.

[3] Venturi V, Keel C (2016). Signaling in the rhizosphere. Trends Plant Sci.

[4] Pawlowski K, Bisseling T (1996). Rhizobial and actinorhizal symbioses: what are the shared features?. Plant Cell.

[5] Brewin NJ (2004). Plant cell wall remodelling in the Rhizobium-legume symbiosis. Crit Rev Plant Sci.

[6] Deakin WJ, Broughton WJ (2009). Symbiotic use of pathogenic strategies: rhizobial protein secretion systems. Nat Rev Microbiol.

[7] Downie JA (2010). The roles of extracellular proteins, polysaccharides and signals in the interactions of rhizobia with legume roots. FEMS Microbiol Rev.

[8] Oldroyd GE (2013). Speak, friend, and enter: signalling systems that promote beneficial symbiotic associations in plants. Nat Rev Microbiol.

[9] Hocher V, Ngom M, Carré-Mlouka A, Tisseyre P, Gherbi H, Svistoonoff S (2019). Signalling in actinorhizal root nodule symbioses. Antonie Van Leeuwenhoek.

[10] Auguy F, Abdel-Lateif K, Doumas P, Badin P, Guerin V, Bogusz D, Hocher V (2011). Activation of the isoflavonoid pathway in actinorhizal symbioses. Funct Plant Biol.

[11] Abdel-Lateif K, Bogusz D, Hocher V (2012). The role of flavonoids in the establishment of plant roots endosymbioses with arbuscular mycorrhiza fungi, rhizobia and Frankia bacteria. Plant Signal Behav.

[12] Popovici J, Comte G, Bagnarol É, Alloisio N, Fournier P, Bellvert F (2010). Differential effects of rare specific flavonoids on compatible and incompatible strains in the Myrica gale-Frankia actinorhizal symbiosis. Appl Environ Microbiol.

[13] Popovici J, Walker V, Bertrand C, Bellvert F, Fernandez MP, Comte G (2011). Strain specificity in the Myricaceae-Frankia symbiosis is correlated to plant root phenolics. Funct Plant Biol.

[14] Benoit LF, Berry AM (1997). Flavonoid-like compounds from seeds of red alder (Alnus rubra) influence host nodulation by Frankia (Actinomycetales). Physiol Plant.

[15] Normand P, Lapierre P, Tisa LS, Gogarten JP, Alloisio N, Bagnarol E (2007). Genome characteristics of facultatively symbiotic Frankia sp. strains reflect host range and host plant biogeography. Genome Res.

[16] Tisa LS, Oshone R, Sarkar I, Ktari A, Sen A, Gtari M (2016). Genomic approaches toward understanding the actinorhizal symbiosis: an update on the status of the Frankia genomes. Symbiosis.

[17] Ktari A, Nouioui I, Furnholm T, Swanson E, Ghodhbane-Gtari F, Tisa LS, Gtari M Permanent draft genome sequence of Frankia sp. NRRL B-16219 reveals the presence of canonical nod genes, which are highly homologous to those detected in Candidatus Frankia Dg1 genome. Stand Genomic Sci. 2017b;12(1):1-10.

[18] Gueddou A, Sarker I, Sen A, Ghodhbane-Gtari F, Benson DR, Armengaud J, Gtari M (2022). Effect of actinorhizal root exudates on the proteomes of Frankia soli NRRL B-16219, a strain colonizing the root tissues of its actinorhizal host via intercellular pathway. Res Microbiol.

[19] Gherbi H, Markmann K, Svistoonoff S, Estevan J, Autran D, Giczey G (2008). SymRK defines a common genetic basis for plant root endosymbioses with arbuscular mycorrhiza fungi, rhizobia, and Frankia bacteria. Proc Natl Acad Sci.

[20] Hocher V, Alloisio N, Auguy F, Fournier P, Doumas P, Pujic P (2011). Transcriptomics of actinorhizal symbioses reveals homologs of the whole common symbiotic signaling cascade. Plant Physiol.

[21] Maillet F, Poinsot V, André O, Puech-Pagès V, Haouy A, Gueunier M (2011). Fungal lipochitooligosaccharide symbiotic signals in arbuscular mycorrhiza. Nature.

[22] Oldroyd GE (2001). Dissecting symbiosis: developments in Nod factor signal transduction. Ann Bot.

[23] Willmann R, Lajunen HM, Erbs G, Newman MA, Kolb D, Tsuda K, Nürnberger T (2011). Arabidopsis lysin-motif proteins LYM1 LYM3 CERK1 mediate bacterial peptidoglycan sensing and immunity to bacterial infection. Proc Natl Acad Sci.

[24] Kouzai Y, Mochizuki S, Nakajima K, Desaki Y, Hayafune M, Miyazaki H (2014). Targeted gene disruption of OsCERK1 reveals its indispensable role in chitin perception and involvement in the peptidoglycan response and immunity in rice. Mol Plant Microbe Interact.

[25] Ao Y, Li Z, Feng D, Xiong F, Liu J, Li JF (2014). Os CERK 1 and Os RLCK 176 play important roles in peptidoglycan and chitin signaling in rice innate immunity. Plant J.

[26] Lopez-Gomez M, Sandal N, Stougaard J, Boller T (2012). Interplay of flg22-induced defence responses and nodulation in Lotus japonicus. J Exp Bot.

[27] Thor K, Peiter E (2014). Cytosolic calcium signals elicited by the pathogen-associated molecular pattern flg22 in stomatal guard cells are of an oscillatory nature. New Phytol.

[28] Keinath NF, Waadt R, Brugman R, Schroeder JI, Grossmann G, Schumacher K, Krebs M (2015). Live cell imaging with R-GECO1 sheds light on flg22-and chitin-induced transient [Ca2+] cyt patterns in Arabidopsis. Mol Plant.

[29] Albert I, Zhang L, Bemm H, Nürnberger T (2019). Structure-function analysis of immune receptor at RLP23 with its ligand nlp20 and coreceptors at SOBIR1 and At BAK1. Mol Plant Microbe Interact.

[30] Zipfel C, Oldroyd GE (2017). Plant signalling in symbiosis and immunity. Nature.

[31] Nelson MS, Sadowsky MJ (2015). Secretion systems and signal exchange between nitrogen-fixing rhizobia and legumes. Front Plant Sci.

[32] Lucke M, Correa MG, Levy A (2020). The role of secretion systems, effectors, and secondary metabolites of beneficial rhizobacteria in interactions with plants and microbes. Front Plant Sci.

[33] Pujic P, Alloisio N, Fournier P, Roche D, Sghaier H, Miotello G (2019). Omics of the early molecular dialogue between Frankia alni and Alnus glutinosa and the cellulase synton. Environ Microbiol.

[34] Ktari A, Gueddou A, Nouioui I, Miotello G, Sarkar I, Ghodhbane-Gtari F (2017). Host plant compatibility shapes the proteogenome of Frankia coriariae. Front Microbiol.

[35] Wang G, Chen H, Xia Y, Cui J, Gu Z, Song Y (2013). How are the non-classically secreted bacterial proteins released into the extracellular milieu?. Curr Microbiol.

[36] Armengaud J, Christie-Oleza JA, Clair G, Malard V, Duport C (2012). Exoproteomics: exploring the world around biological systems. Expert Rev Proteomics.

[37] Desvaux M, Hébraud M, Talon R, Henderson IR (2009). Secretion and subcellular localizations of bacterial proteins: a semantic awareness issue. Trends Microbiol.

[38] Maffei B, Francetic O, Subtil A (2017). Tracking proteins secreted by bacteria: what's in the toolbox?. Front Cell Infect Microbiol.

[39] Sauvage S, Hardouin J (2020). Exoproteomics for better understanding Pseudomonas aeruginosa virulence. Toxins.

[40] von Tils D, Blädel I, Schmidt MA, Heusipp G (2012). Type II secretion in Yersinia—a secretion system for pathogenicity and environmental fitness. Front Cell Infect Microbiol.

[41] Cabrita P, Trigo MJ, Ferreira RB, Brito L (2014). Is the exoproteome important for bacterial pathogenesis?. Lessons learned from interstrain exoprotein diversity in Listeria monocytogenes grown at different temperatures. Omics.

[42] Mulcahy ME, McLoughlin RM (2016). Host-bacterial crosstalk determines Staphylococcus aureus nasal colonization. Trends Microbiol.

[43] Savinova OS, Glazunova OA, Moiseenko KV, Begunova AV, Rozhkova IV, Fedorova TV (2021). Exoproteome analysis of antagonistic interactions between the probiotic bacteria Limosilactobacillus reuteri LR1 and Lacticaseibacillus rhamnosus F and multidrug resistant strain of Klebsiella pneumonia. Int J Mol Sci.

[44] Ciprandi A, Da Silva WM, Santos AV, de Castro Pimenta AM, Carepo MSP, Schneider MPC (2013). Chromobacterium violaceum: important insights for virulence and biotechnological potential by exoproteomic studies. Curr Microbiol.

[45] Biagini M, Bagnoli F, Norais N (2017). Surface and Exoproteomes of Gram-Positive Pathogens for Vaccine Discovery. In: Protein and Sugar Export and Assembly in Gram-positive Bacteria.

[46] Dou D, Zhou J-MM (2012). Phytopathogen efectors subverting host immunity: Diferent foes, similar battleground. Cell Host Microbe.

[47] Khatabi B, Gharechahi J, Ghaffari MR, Liu D, Haynes PA, McKay MJ (2019). Plant-microbe symbiosis: what has proteomics taught us?. Proteomics.

[48] Lidbury ID, Raguideau S, Liu S, Murphy AR, Stark R, Borsetto C Meta-exoproteomics identifies active plant-microbe interactions operating in the rhizosphere. bioRxiv. 2021-09.

[49] Broughton WJ, Dilworth M (1971). Control of leghaemoglobin synthesis in snake beans. Biochem J.

[50] Gtari M, Ghodhbane-Gtari F, Nouioui I (2020). Frankia soli sp. nov, an actinobacterium isolated from soil beneath Ceanothus jepsonii. Int J Syst Evol Microbiol.

[51] Liu H, Sadygov RG, Yates JR (2004). A model for random sampling and estimation of relative protein abundance in shotgun proteomics. Anal Chem.

[52] Zivanovic Y, Armengaud J, Lagorce A, Leplat C, Guérin P, Dutertre M (2009). Genome analysis and genome-wide proteomics of Thermococcus gammatolerans, the most radioresistant organism known amongst the Archaea. Genome Biol.

[53] Paoletti AC, Parmely TJ, Tomomori-Sato C, Sato S, Zhu D, Conaway RC (2006). Quantitative proteomic analysis of distinct mammalian Mediator complexes using normalized spectral abundance factors. Proc Natl Acad Sci.

[54] Hiller K, Grote A, Scheer M, Münch R, Jahn D (2004). PrediSi: prediction of signal peptides and their cleavage positions. Nucleic Acids Res.

[55] Teufel F, Almagro Armenteros JJ, Johansen AR, Gíslason MH, Pihl SI, Tsirigos KD (2022). SignalP 6.0 predicts all five types of signal peptides using protein language models. Nat Biotechnol.

[56] Bendtsen JD, Nielsen H, Widdick D, Palmer T, Brunak S (2005). Prediction of twin-arginine signal peptides. BMC Bioinformatics.

[57] Bendtsen JD, Kiemer L, Fausboll A, Brunak S (2005). Non-classical protein secretion in bacteria. BMC Microbiol.

[58] Wang Y, Wei X, Bao H, Liu SL (2014). Prediction of bacterial type IV secreted effectors by C-terminal features. BMC Genomics.

[59] Eichinger V, Nussbaumer T, Platzer A, Jehl MA, Arnold R, Rattei T (2016). EffectiveDB—updates and novel features for a better annotation of bacterial secreted proteins and Type III, IV, VI secretion systems. Nucleic Acids Res.

[60] de Jong A, Kuipers OP, Kok J (2022). FUNAGE-Pro: comprehensive web server for gene set enrichment analysis of prokaryotes. Nucleic Acids Res.

[61] Rubiano-Labrador C, Bland C, Miotello G, Armengaud J, Baena S (2015). Salt stress induced changes in the exoproteome of the halotolerant bacterium Tistlia consotensis deciphered by proteogenomics. PLoS One.

[62] Madeira JP, Alpha-Bazin BM, Armengaud J, Duport C (2017). Methionine residues in exoproteins and their recycling by methionine sulfoxide reductase AB serve as an antioxidant strategy in Bacillus cereus. Front Microbiol.

[63] Shockman GD, Daneo-Moore L, Kariyama R, Massidda O (1996). Bacterial walls, peptidoglycan hydrolases, autolysins, and autolysis. Microb Drug Resist.

[64] Lopez MF, Fontaine MS, Torrey JG (1984). Levels of trehalose and glycogen in Frankia sp. HFPArI3 (Actinomycetales). Can J Microbiol.

[65] Murry MA, Fontaine MS, Torrey JG (1984). Growth kinetics and nitrogenase induction in Frankia sp. HFPArI 3 grown in batch culture. Plant Soil.

[66] Schwencke J (1991). Rapid, exponential growth and increased biomass yield of some Frankia strains in buffered and stirred mineral medium (BAP) with phosphatidyl choline. In: Nitrogen Fixation: Proceedings of the Fifth International Symposium on Nitrogen Fixation with Non-Legumes, Florence, Italy, 10-14 September 1990. Springer Netherlands.

[67] Burggraaf AJP, Shipton WA (1983). Studies on the growth of Frankia isolates in relation to infectivity and nitrogen fixation (acetylene reduction). Can J Bot.

[68] Newcomb W, Peterson RL, Callaham D, Torrey JG (1978). Structure and host-actinomycete interactions in developing root nodules of Comptonia peregrina. Can J Bot.

[69] Mastronunzio JE, Huang Y, Benson DR (2009). Diminished exoproteome of Frankia spp. in culture and symbiosis. Appl Environ Microbiol.

[70] Benoist PATRICK, Müller A, Diem HG, Schwencke J (1992). High-molecular-mass multicatalytic proteinase complexes produced by the nitrogen-fixing actinomycete Frankia strain BR. J Bacteriol.

[71] Cheggour A, Fanuel L, Duez C, Joris B, Bouillenne F, Devreese B (2000). The dppA gene of Bacillus subtilis encodes a new d-aminopeptidase. Mol Microbiol.

[72] van Bergeijk DA, Terlouw BR, Medema MH, van Wezel GP (2020). Ecology and genomics of Actinobacteria: new concepts for natural product discovery. Nat Rev Microbiol.

[73] Rigali S, Titgemeyer F, Barends S, Mulder S, Thomae AW, Hopwood DA, Van Wezel GP (2008). Feast or famine: the global regulator DasR links nutrient stress to antibiotic production by Streptomyces. EMBO Rep.

[74] Planas A (2022). Peptidoglycan Deacetylases in Bacterial Cell Wall Remodeling and Pathogenesis. Curr Med Chem.

[75] Aspell T, Khemlani AHJ, Tsai CJY, Loh JMS, Proft T (2023). The Cell Wall Deacetylases Spy1094 and Spy1370 Contribute to Streptococcus pyogenes Virulence. Microorganisms.

[76] Geelen D, Leyman B, Mergaert P, Klarskov K, Van Montagu M, Geremia R, Holsters M (1995). NodS is an S-adenosyl-l-methionine-dependent methyltransferase that methylates chitooligosaccharides deacetylated at the non-reducing end. Mol Microbiol.

[77] Grohmann E, Christie PJ, Waksman G, Backert S (2018). Type IV secretion in Gram-negative and Gram-positive bacteria. Mol Microbiol.

[78] Costa TR, Harb L, Khara P, Zeng L, Hu B, Christie PJ (2021). Type IV secretion systems: advances in structure, function, and activation. Mol Microbiol.

[79] Hubber A, Vergunst AC, Sullivan JT, Hooykaas PJ, Ronson CW (2004). Symbiotic phenotypes and translocated effector proteins of the Mesorhizobium loti strain R7A VirB/D4 type IV secretion system. Mol Microbiol.

[80] Sugawara M, Epstein B, Badgley BD, Unno T, Xu L, Reese J, Sadowsky MJ (2013). Comparative genomics of the core and accessory genomes of 48 Sinorhizobium strains comprising five genospecies. Genome Biol.

[81] Gómez-Gómez L, Boller T (2000). FLS2: an LRR receptor-like kinase involved in the perception of the bacterial elicitor flagellin in Arabidopsis. Mol Cell.

[82] Silipo A, Molinaro A, Sturiale L, Dow JM, Erbs G, Lanzetta R (2005). The Elicitation of Plant Innate Immunity by Lipooligosaccharide of Xanthomonas campestris. J Biol Chem.

[83] Zipfel C, Kunze G, Chinchilla D, Caniard A, Jones JD, Boller T, Felix G (2006). Perception of the bacterial PAMP EF-Tu by the receptor EFR restricts Agrobacterium-mediated transformation. Cell.

[84] Meneses N, Taboada H, Dunn MF, Vargas MDC, Buchs N, Heller M, Encarnación S (2017). The naringenin-induced exoproteome of Rhizobium etli CE3. Arch Microbiol.

[85] Aslam SN, Erbs G, Morrissey KL, NEWMAN MA, Chinchilla D, Boller T (2009). Microbe-associated molecular pattern (MAMP) signatures, synergy, size and charge: influences on perception or mobility and host defence responses. Mol Plant Pathol.

[86] Soto MJ, Domínguez-Ferreras A, Pérez-Mendoza D, Sanjuán J, Olivares J (2009). Mutualism versus pathogenesis: the give-and-take in plant-bacteria interactions. Cell Microbiol.

[87] Chabaud M, Gherbi H, Pirolles E, Vaissayre V, Fournier J, Moukouanga D (2016). Chitinase-resistant hydrophilic symbiotic factors secreted by Frankia activate both Ca2+ spiking and NIN gene expression in the actinorhizal plant Casuarina glauca. New Phytol.

